# Impact of stent edge dissection detected by optical coherence tomography after current-generation drug-eluting stent implantation

**DOI:** 10.1371/journal.pone.0259693

**Published:** 2021-11-04

**Authors:** Hiroyuki Jinnouchi, Kenichi Sakakura, Tomonobu Yanase, Yusuke Ugata, Takunori Tsukui, Yosuke Taniguchi, Kei Yamamoto, Masaru Seguchi, Hiroshi Wada, Hideo Fujita

**Affiliations:** Division of Cardiovascular Medicine, Saitama Medical Center, Jichi Medical University, Saitama, Japan; Baylor Scott and White, Texas A&M College of Medicine, UNITED STATES

## Abstract

**Background:**

Stent edge dissection (SED) is a well-known predictor of worse clinical outcomes. However, impact of SED after current-generation drug-eluting stent (DES) implantation remains unknown since there was no study using only current-generation DES to assess impact of SED. This study aimed to investigate a relationship between SED detected by optical coherence tomography (OCT) and clinical outcomes after current-generation DES implantation.

**Methods:**

This study enrolled 175 patients receiving OCT after current-generation DES implantation. The SED group was compared with the non-SED group in terms of the primary study endpoints which was the cumulative incidence of major adverse cardiac event (MACE) composed of cardiac death, target vessel myocardial infarction (TV-MI), and clinically-driven target lesion revascularization (CD-TLR).

**Results:**

Of 175 patients, SED detected by OCT was observed in 32 patients, while 143 patients did not show SED. In the crude population, the SED group showed a significantly higher incidence of CD-TLR, definite stent thrombosis, TV-MI and cardiac death relative to the non-SED group. After adjustment by an inverse probability weighted methods, the SED group showed a significantly higher incidence of MACE compared with the non-SED group (hazard ratio 3.43, 95% confidence interval 1.09–10.81, p = 0.035). Fibrocalcific or lipidic plaques, greater lumen eccentricity, and stent-oversizing were the predictors of SED.

**Conclusions:**

SED detected by OCT after the current-generation DES implantation led to unfavorable outcomes. Aggressive post-dilatation around the stent edge might worse clinical outcomes due to SED, although achievement of optimal stent expansion is strongly encouraged to improve clinical outcomes.

## Introduction

Stent implantation can result in vessel wall injury between the edge of a stent and the adjacent vessel wall [1, 2]. It has been previously reported that residual stent edge dissection (SED) has been associated with a high rate of stent thrombosis and major adverse cardiac events (MACE) [3–5]. SED without flow limitation can be treated according to the operator’s discretion, since there are no clear criteria to judge whether SED should be covered by an additional stent. Angiography and intravascular ultrasound (IVUS) have been used to diagnose SED during procedures of percutaneous coronary intervention (PCI) [3–5]. Previous data showing a relationship between SED and worse clinical outcomes has been demonstrated mainly by angiography or IVUS [3–5]. Optical coherence tomography (OCT) is designed to detect intracoronary structures such as luminal surface and intimal components in detail with a high resolution of 10–20 μm [6]. OCT has enabled the detection of SED that IVUS fails to reveal [7, 8]. Recently, several studies showed that SED detected by OCT was associated with worse clinical outcomes, although there is still a controversy as to whether SED detected by OCT affects clinical outcomes [9–14].

Regardless of imaging modalities such as IVUS or OCT, there was no study that evaluated clinical outcomes of SED after only current-generation DES implantation. The current-generation DES has provided a different performance from bare metal stent (BMS) and first-generation DES since it improved safety and feasibility compared with BMS or first-generation DES [15, 16]. It remains unknown how SED detected by OCT affects clinical outcomes in the current-generation DES era. The purpose of this study was to investigate whether SED detected by OCT affected clinical outcomes after current-generation DES implantation.

## Materials and methods

### Study population

This study was a single-center, retrospective observational study at Saitama Medical Center, Jichi Medical University. Between April 2010 and March 2020, the consecutive patients undergoing PCI were reviewed. The inclusion criteria were as follows: 1) OCT or optical frequency domain imaging (OFDI) were performed, and 2) current-generation DESs were implanted. The exclusion criteria were as follows: 1) stent was not required during the procedure, 2) BMS or first-generation DES were implanted, 3) final OCT or OFDI images after the procedures were not available, and 4) quality of images was poor to analyze. When a patient received OCT procedures more than once during the study period, only the initial procedure was included. Those patients were divided into the SED and non-SED groups according to the presence of SED detected by OCT. SED detected by OCT was defined as a disruption of the vessel luminal surface with flap at an adjacent site to the stent edge (< 5mm). The strategy of procedures was dependent on operators using OCT assessment. If necessary, the lesion preparation such as pre-dilatation, aspiration and rotational atherectomy was performed before stenting. Pre-dilatation was considered when stent underexpansion and the difficulty of device derivability were expected, or when pre-dilatation makes it easy to perform the next procedure for any reasons. Aspiration was performed when an obvious thrombus by angiography was observed, or when the effectiveness of aspiration was expected. Rotational atherectomy was required when heavily calcified lesions by angiography or intra-coronary imaging such as IVUS or OCT was observed, or unsuccessful balloon dilatation or unsuccessful balloon delivery occurred due to calcification. This study was approved by the institutional review board of Saitama Medical Center, Jichi Medical University (S20-124), and written informed consent was waived because of the retrospective study design. Follow-up data until August 2020 were obtained from a review of hospital records based on clinic visits. Saitama Medical Center, Jichi Medical University is a local core hospital. The annual average number of PCI was approximately 400 to 800 cases a year during this study-period. There are two catheter rooms and at least several interventional cardiologists performed the PCI-procedures in this hospital, although they were not consistent for this study-period from 2010 to 2020.

The primary study endpoints were the cumulative incidence of MACE which were composed of cardiac death, target vessel myocardial infarction (TV-MI), and clinically-driven target lesion revascularization (CD-TLR). All-cause of death, stent thrombosis, and target vessel revascularization (TVR) were also evaluated in this study. Definitions of clinical endpoints were based on the Academic Research Consortium (ARC) [17]. Cardiac death was defined as any death due to a proximate cardiac cause, unwitnessed death or death of unknown cause, and all procedure-related death. TV-MI was defined as MI in the treated vessel [17]. TLR was defined as any revascularization (either repeated PCI or coronary artery bypass graft [CABG]) within the stent and 5mm proximally and distally to the stent [17]. TVR was defined as any revascularization (either repeated PCI or repeated CABG) of the target vessel. Revascularization was considered clinically driven if associated with any of the following: (1) positive functional ischemia study, (2) ischemic symptoms and angiographic diameter stenosis ≥50%, and (3) angiographic diameter stenosis ≥70% without angina or positive functional study [17, 18]. The diagnostic certainty of stent thrombosis, i.e., definite or probable, was evaluated according to the ARC definition [17].

### OCT image acquisition

We performed OCT using one of the following systems: M2 OCT system (Light Lab Imaging, Westford, MA, USA; C7XR Fourier-Domain System (St Jude Medical, St Paul, MN, USA), ILUMIEN (St Jude Medical, St Paul, MN, USA), OPTIS (Abbott Vascular, Santa Clara, CA, USA), and LUNAWAVE (Terumo, Tokyo, Japan). Motorized pullback OCT imaging was performed at a rate of 1.0 mm/s through the stent. Images were acquired at 15.6 frames/s and digitally archived. C7XR, ILUMIEN, and OPTIS system were acquired automatically at a pullback rate of 20 mm/s (100 frames/s) or 36 mm/s (180 frames/s) and that of OFDI at a pullback rate of 20 mm/s (160 frames/s). All images were stored digitally and analyzed offline by the LightLab OCT imaging proprietary software (LightLab Imaging), ILUMIEN/OPTIS software (Abbott Vascular, Santa Clara, CA, USA), or Terumo software. Procedure details using each modality have been previously described [19].

### OCT image analysis

All OCT images were analyzed based on conventional definitions reported in expert consensus OCT documents [20, 21]. Using automated contour-detection software (OCT system, St Jude Medical or Abbott Vascular, OFDI system, Terumo), stent and lumen cross-sectional areas were measured within the stent and 5mm proximally and distally to the stent. Proximal and distal reference lumen was defined as the largest outside of the stents. In-stent lumen expansion was defined as the percentage of in-stent lumen area/ the average reference lumen area.

**Fig 1:**
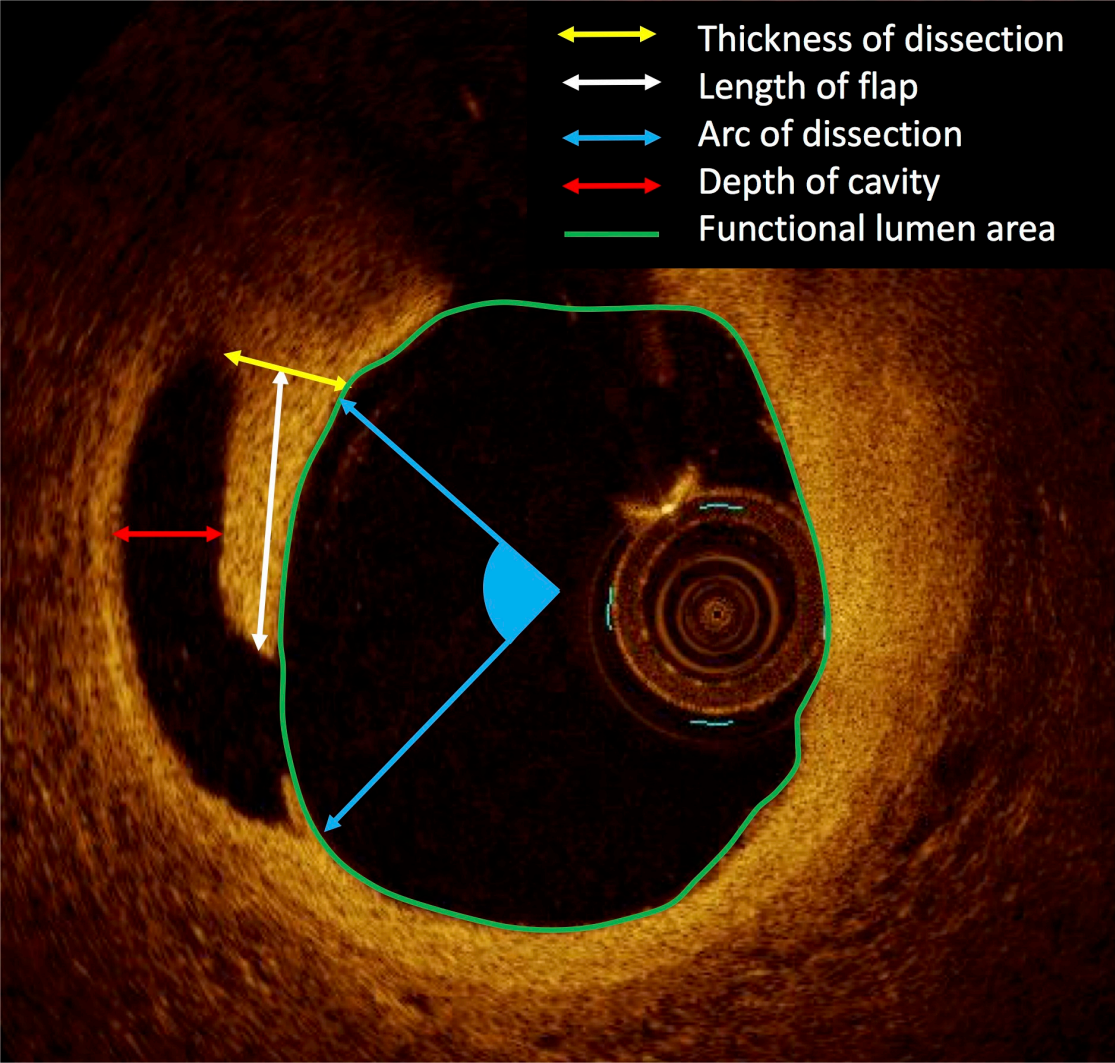
Measurement of stent edge dissection.

Stent border was defined as the first and last cross-sections of the stented segment where struts could be seen in all 4 quadrants [13]. Stent border area is stent area at the stent border. Peri-stent lumen was defined as the first frame following a stent where any struts could not be seen [13]. At peri-stent lumen, maximum and minimum lumen diameter were measured. Stent-oversizing index was defined as stent border area / reference area. Lumen eccentricity was defined as (maximum lumen diameter–minimum lumen diameter)/ maximum lumen diameter [13]. Type of plaque was categorized as the following 4: 1) normal intima which is characterized by intima with a thickness of < 250μm; 2) lipidic plaque; 3) fibrocalcific plaque, and 4) fibrous plaque [21, 22]. Lipidic plaque was defined as a plaque with a signal poor region with diffuse borders. Fibrocalcific plaque was defined as a plaque with a signal-poor or heterogeneous region and a sharply delineated border. Fibrous plaque was defined as a plaque showing homogeneous and rich OCT signal. If lipidic and fibrocalcific plaque were observed in the same cross-sectional images, either the dominant type was selected. The progressive atherosclerotic lesion was defined as lipidic or fibrocalcific lesions. Most affected cross-sections having the largest dissection were selected to be assessed. The length of the flap was defined as the distance between the tip and bottom of the flap (Fig 1). The thickness of the flap was measured at the bottom of the flap. Arc of dissection was defined as the angle of dissection. The depth of the cavity was defined as a maximal distance in an empty space underneath a flap. The functional lumen area was defined as a lumen area without a flap and space of the cavity. Depth of vessel injury was assessed as the following 3 categories; 1) intima, 2) media, and 3) adventitia.

### Statistical analysis

Data are presented as values and percentages, mean±SD, or median (interquartile range). Categorical variables were compared between the 2 groups with Fisher’s exact test. Continuous variables were compared between groups using the unpaired t-test or the Mann-Whitney U test, based on the data distribution. For edge-level analysis, the generalized estimated equation (GEE) methods was used. Continuous variables were tested by the GEE method with gamma with the log-link based on the data distribution. Categorical data were tested by the GEE method with an ordinal logistic model. For each endpoint, the cumulative incidence probability through the study-period was estimated using the Kaplan-Meier method. The statistical analysis to adjust the background is necessary since this is a retrospective study. In this study including the small number, the inverse probability treatment weighting (IPTW) using the propensity score is chosen to retain all the patient data since propensity score matching reduces the population after matching patients [23]. Hazard ratio (HR) of SED versus non-SED for MACE was estimated through IPTW Cox model with covariate set. Weights for the IPW methods were estimated through a logistic model for probabilities of SED conditional on covariate set (age, sex, diabetes mellitus, hemodialysis, ejection fraction ≤40%, AHA/ACC type B2 or C). Weights did not highly vary among patients (range: 1.1 to 8.0). Multivariate logistic regression analysis was performed to identify independent predictors for SED. In the multivariate model, variables with P<0.10 in Table 2 were used. If clinically similar variables remained, we selected the variable that we considered to be more clinically relevant. Statistical analysis was performed using JMP version 10, SPSS version 24 and STATA/SE version 15.1. Two-sided P<0.05 was considered to indicate statistical significance.

## Results

### Patient and lesion characteristics

**Fig 2:**
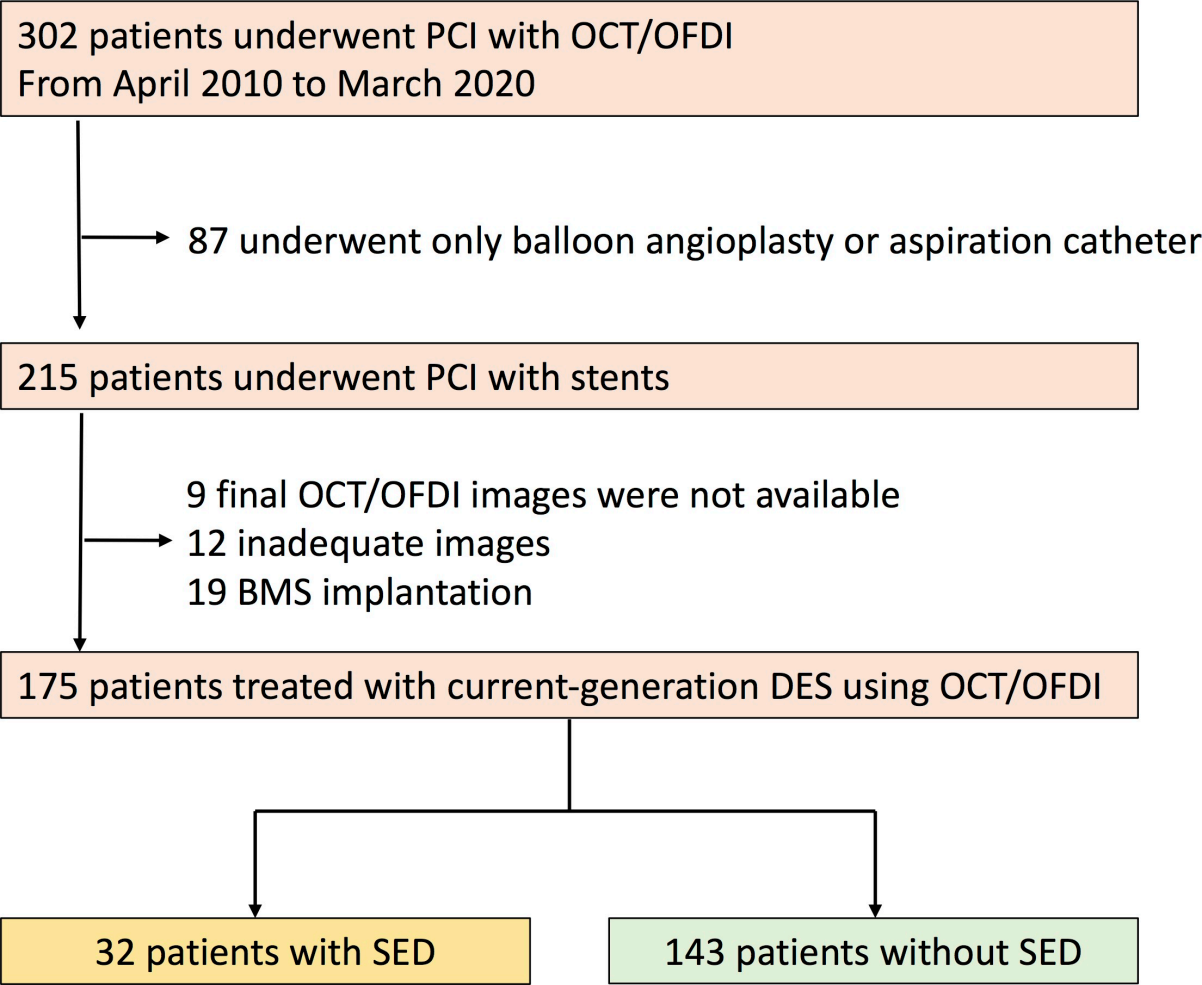
Study flow chart. DES, drug-eluting stent; OCT, optical coherence tomography; OFDI, optical frequency domain imaging; PCI, percutaneous coronary intervention; SED, stent edge dissection.

During this study period, 302 patients underwent PCI with OCT. Finally, of these patients, 175 patients who were treated with current-generation DESs and underwent post-procedure imaging by OCT were enrolled in this study (Fig 2). Those patients were divided into 2 groups: 1) the SED group (32 patients with 32 lesions) and 2) the non-SED group (143 patients with 143 lesions). The patient characteristics were comparable except for acute coronary syndrome, which was higher in the SED group relative to the non-SED group (Table 1). The median follow-up period did not significantly differ between the SES and non-SED groups [856 days (234–2098) vs. 870 days (203–1534), respectively, p = 0.37)]. There were no significant differences between the 2 groups in lesion characteristics except for the usage of aspiration for patients with ACS, which was higher in the SED group relative to the non-SED group (Table 2).

**Table 1:** Patient characteristics.

	Overall	SED	Non-SED	p-value
	(N = 175)	(N = 32)	(N = 143)	
Age, years	69.3±9.2	68.1± 9.2	69.6± 9.2	0.40
Male	139 (79.4)	24 (75.0)	115 (80.4)	0.48
Risk Factor				
Hypertension	118 (67.4)	19 (59.4)	99 (69.2)	0.30
Hyperlipidemia	102 (58.3)	20 (62.5)	82 (57.3)	0.69
Diabetes	72 (41.1)	13 (40.6)	59 (41.3)	1.00
Current smoker	33 (18.9)	8 (25.0)	25 (17.5)	0.33
Family History of CAD	32 (18.3)	5 (15.6)	27 (18.9)	0.80
Past medical history				
Previous MI	34 (19.4)	6 (18.8)	28 (19.6)	1.00
Previous PCI	55 (31.4)	8 (25.0)	47 (32.9)	0.53
Previous CABG	3 (1.7)	0 (0.0)	3 (2.1)	1.00
Hemodialysis	10 (5.7)	4 (12.5)	6 (4.2)	0.09
Presentation				0.007
Acute coronary syndrome	46 (26.3)	15 (46.9)	31 (21.7)	
Stable angina	129 (73.7)	17 (53.1)	112 (78.3)	
eGFR, ml/min/1.73m^2^	70.2 (60.2–83.7)	72.6 (61.4–94.4)	70.2 (59.9–80.6)	0.31
Ejection fraction, %	60.8± 11.9	57.5± 13.2	61.5± 11.5	0.09
Body mass index, kg/m^2^	24.3± 3.5	23.8± 4.0	24.4± 3.4	0.35
Number of disease				0.85
1 vessel	110 (62.9)	20 (62.5)	90 (62.9)	
2 vessels	55 (31.4)	11 (34.4)	44 (30.8)	
3 vessels	10 (5.7)	1 (3.1)	9 (6.3)	
Multi-vessel disease	65 (37.1)	12 (37.5)	53 (37.1)	1.00
Follow-up period, days	870 (203–1588)	856 (234–2098)	870 (203–1534)	0.37

Values are presented as median (interquartile range), or n (%) for categorical variables. CABG, coronary artery bypass graft; CAD, coronary artery disease; eGFR, estimated glomerular filtration rate; MI, myocardial infarction; PCI, percutaneous coronary intervention; SED, stent edge dissection.

**Table 2:** Lesion and procedural characteristics and OCT analysis.

	Overall	SED	Non-SED	p-value
	(N = 175)	(N = 32)	(N = 143)	
Culprit lesions				0.96
RCA	42 (24.0)	8 (25.0)	34 (23.8)	
LAD	98 (56.0)	19 (59.4)	79 (55.2)	
LCX	32 (18.3)	5 (15.6)	27 (18.9)	
LMT	2 (1.1)	0 (0.0)	2 (1.4)	
SVG	1 (0.6)	0 (0.0)	1 (0.7)	
AHA/ACC lesion classification				0.03
A	50 (28.6)	3 (9.4)	47 (32.9)	
B1	48 (27.4)	12 (37.5)	36 (25.2)	
B2	20 (11.4)	5 (15.6)	15 (10.5)	
C	57 (32.6)	12 (37.5)	45 (31.5)	
Type of stent				0.57
DP-EES	106 (60.6)	21 (65.6)	85 (59.4)	
R-ZES	31 (17.7)	7 (21.9)	24 (16.8)	
BP-EES	23 (13.1)	3 (9.4)	20 (14.0)	
BP-SES	15 (8.6)	1 (3.1)	14 (9.8)	
Number of stents				
1 stent	162 (92.6)	27 (84.4)	135 (94.4)	0.0504
2 stents	12 (6.9)	4 (12.5)	8 (5.6)	
3 stents	1 (0.6)	1 (3.1)	0 (0.0)	
Stent diameter, mm	3.0 (2.5–3.0)	3.0 (2.7–3.0)	3.0 (2.5–3.0)	0.57
Total stent length, mm	20 (16–28)	22 (18–28)	20 (16–28)	0.37
Lesion preparation				
Pre-dilatation	116 (66.3)	20 (62.5)	96 (67.1)	0.68
Aspiration	19 (10.9)	8 (25.0)	11 (7.7)	0.009
Rotational atherectomy	7 (4.0)	1 (3.1)	6 (4.2)	1.00
Post-balloon dilatation	77 (44.0)	18 (56.3)	59 (41.3)	0.17
Direct stenting	63 (36.0)	14 (43.8)	49 (34.3)	0.32
Overlapping stent	13 (7.4)	5 (15.6)	8 (5.6)	0.06
OCT/OFDI analysis				
Proximal reference, mm	6.4 (4.8–8.3)	6.3 (4.3–8.4)	6.4 (4.8–8.3)	0.49
Distal reference, mm	4.7 (3.5–5.9)	4.2 (3.3–5.8)	4.8 (3.6–6.1)	0.32
Mean reference, mm	5.7 (4.3–7.2)	5.9 (3.8–6.6)	5.7 (4.4–7.4)	0.60
Minimal stent area, mm^2^	5.0 (3.9–6.1)	5.3 (3.6–6.4)	5.0 (3.9–5.9)	0.64
Percent of stent expansion, %	89.8 (78.7–103.4)	95.4 (87.3–100.7)	87.0 (77.0–104.8)	0.051

Values are presented as median (interquartile range), or n (%) for categorical variables. ACC, American College of Cardiology; AHA, American Heart Association; BP, biodegradable polymer; DP, durable polymer; EES, everolimus-eluting stent; LAD left anterior descending artery; LCX, left circumflex artery; LMT, left main trunk; OCT, optical coherence tomography; OFDI, optical frequency domain imaging; RCA, right coronary artery; SES, sirolimus-eluting stent; SVG, saphenous vein graft.

### OCT assessment

Proximal, distal and mean reference areas, minimal stent area, and percent expansion were not significantly different between the 2 groups (Table 2). Table 3 listed OCT data at stent edges with and without dissection. Edges with dissection showed significantly smaller reference lumen area relative to edges without dissection. Stent-oversizing index, lumen long diameter to short diameter ratio and lumen eccentricity were significantly greater in edges with dissection than without dissection. Prevalence of plaque-type at stent edge was different between edges with and without dissection. Progressive atherosclerotic plaques (i.e., lipidic and fibrocalcific plaques) were more frequently found in edges with dissection.

**Table 3:** OCT/OFDI analysis at stent edges.

	Overall	Edge with dissection	Edge without dissection	p-value
	(N = 345)	(N = 35)	(N = 310)	
Reference lumen area, mm^2^	5.3 (4.0–7.3)	4.3 (3.8–5.7)	5.4 (4.1–7.4)	0.0009
Stent area at stent border, mm^2^	6.1 (4.7–7.8)	6.0 (4.7–7.6)	6.1 (4.6–7.8)	0.82
Lumen area at adjacent site of stent edge, mm^2^	5.7 (4.3–7.7)	5.0 (4.0–6.6)	5.7 (4.3–7.7)	0.07
Stent-oversizing index	1.1 (1.0–1.3)	1.4 (1.1–1.7)	1.1 (1.0–1.3)	<0.0001
Lumen long diameter / lumen short diameter	1.1 (1.1–1.2)	1.2 (1.1–1.3)	1.1 (1.1–1.2)	0.009
Lumen eccentricity	0.12 (0.09–0.17)	0.16 (0.12–0.24)	0.12 (0.09–0.16)	0.002
Type of plaque at stent edge				0.87
Normal	30 (8.7)	0 (0.0)	30 (9.7)	
Fibrous	208 (60.3)	8 (22.9)	200 (64.5)	
Lipidic	64 (18.6)	16 (45.7)	48 (15.5)	
Fibrocalcific	43 (12.5)	11 (31.4)	32 (10.3)	
Progressive atherosclerotic plaque	107 (31.0)	27 (77.1)	80 (25.8)	<0.0001
Location of dissection				
Proximal	-	12 (34.3)	-	
Distal	-	23 (65.7)	-	
Length of flap, mm	-	0.9 (0.4–1.4)	-	
Thickness of flap, mm	-	0.3 (0.2–0.4)	-	
Length of dissection, mm	-	2.4 (1.5–3.4)	-	
Arc of dissection, °	-	45 (32–82)	-	
Depth of cavity, mm	-	0.3 (0.2–0.4)	-	
Functional lumen area, mm^2^	-	3.7 (2.6–5.6)	-	
Depth of dissection				
Intima	-	22 (62.9)	-	
Media	-	13 (37.1)	-	
Adventitia	-	0 (0.0)	-	
Hematoma	-	2 (5.7)	-	

Values are presented as median (interquartile range), or n (%) for categorical variables. Generalized estimating equation (GEE) method with gamma with log-link model and was used for continuous valuables and GEE with ordinal logistic model was used for categorical data. OCT, optical coherence tomography; OFDI, optical frequency domain imaging.

Morphological and quantitative data of SED was shown in Table 3. SED was more frequently observed in distal locations relative to proximal locations (65.7% vs. 34.3%, respectively). SED reached intima in 62.9% and media in 37.1% of cases.

### Clinical outcomes between SED and non-SED groups

**Fig 3:**
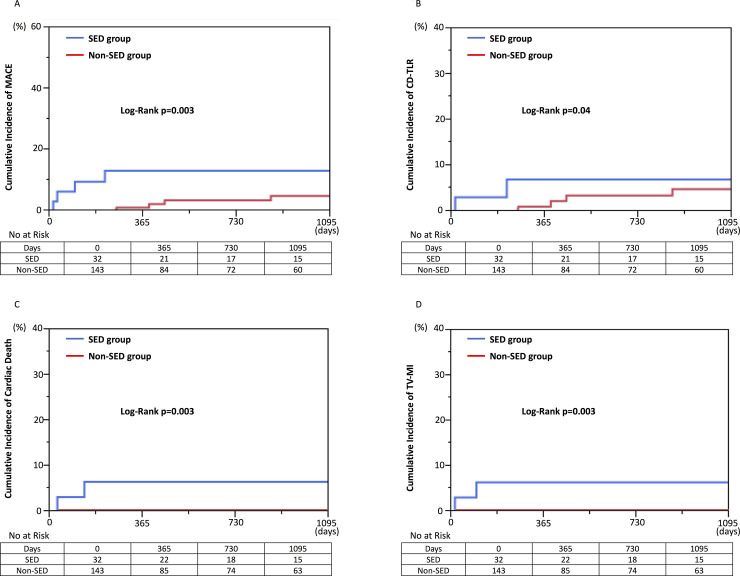
Comparison of clinical outcomes between SED and non-SED groups in the crude population. Kaplan-Meier curve was expressed up to 3 years. P-value was analyzed based on overall periods. (A) major adverse cardiac event (MACE), (B) clinically-driven target lesion revascularization (CD-TLR), (C) cardiac death, and (D) target-vessel myocardial infarction (TV-MI). SED, stent edge dissection.

Table 4 summarized clinical outcomes between the SED and non-SED groups. In the crude population, the incidence of MACE was significantly higher in the SED group compared with the non-SED group (21.9% vs. 4.2%, respectively, p = 0.003) (Fig 3A). After adjustment for baseline characteristics by IPW, SED was significantly associated with increased risks for MACE. There were no significant differences of all cause death, cardiac death and non-cardiac death between the 2 groups. The SED group showed significantly higher incidence of CD-TLR, definite stent thrombosis, TV-MI and cardiac death (15.6% vs. 4.2%, p = 0.04; 3.1% vs. 0.0%, p = 0.04; 6.3% vs. 0.0%, p = 0.003; 6.3% vs. 0.0%, p = 0.003) (Fig 3B–3D).

**Table 4:** Clinical outcomes between SED and non-SED groups.

	Event rate (%)			Crude	IPW Adjusted	
	Overall (n = 175)	SED (n = 32)	Non-SED (n = 143)	p-value	HR (95% CI)	p-value
All cause death	8 (4.6)	3 (9.4)	5 (3.5)	0.27	-	-
Cardiac death	2 (1.1)	2 (6.3)	0 (0.0)	0.003	-	-
Non-cardiac death	6 (3.4)	1 (3.1)	5 (3.5)	0.71	-	-
Target-vessel MI	2 (1.1)	2 (6.3)	0 (0.0)	0.003	-	-
Definite stent thrombosis	1 (0.6)	1 (3.1)	0 (0.0)	0.04	-	-
Probable stent thrombosis	0 (0.0)	0 (0.0)	0 (0.0)	NE	-	-
Any TLR	11 (6.3)	5 (15.6)	6 (4.2)	0.04	-	-
CD-TLR	11 (6.3)	5 (15.6)	6 (4.2)	0.04	-	-
Any TVR	12 (6.9)	5 (15.6)	7 (4.9)	0.07	-	-
CD-TVR	11 (6.3)	5 (15.6)	6 (4.2)	0.04	-	-
MACE	13 (7.4)	7 (21.9)	6 (4.2)	0.003	3.43 (1.09–10.81)	0.035

P-value in the crude-population was analyzed by the log-rank method. CD, clinically-driven; CI, confidence interval; HR, hazard ratio; IPW, inverse probability weighted; MACE, major adverse cardiovascular events; MI, myocardial infarction; NE, not estimable; SED, stent edge dissection; TLR, target lesion revascularization; TVR, target vessel revascularization.

### The predictors for SED

In the analysis of stent edges, 5 edges were excluded since proximal edges were not observed by OCT due to ostium stenting. The independent risk factor for SED was lumen eccentricity (Odds ratio [OR], 1.59; 95% CI: 1.02–2.43, p = 0.03), stent-oversizing index (OR, 1.24; 95% CI: 1.11–1.40, p = 0.0003) and progressive atherosclerotic lesion (OR, 9.58; 95% CI: 4.16–24.66, p<0.0001) (Table 5).

**Table 5:** Predictors for SED.

	Univariable Model		Multivariable Model	
	Odds ratio (95% CI)	p-value	Odds ratio (95% CI)	p-value
Oversizing index (increase 0.1)	1.22 (1.11–1.35)	<0.0001	1.24 (1.11–1.40)	0.0003
Lumen eccentricity (increase 0.1)	1.85 (1.26–2.72)	0.002	1.59 (1.02–2.43)	0.03
Progressive atherosclerotic lesion	9.70 (4.42–23.67)	<0.0001	9.58 (4.16–24.66)	<0.0001

CI, confidence interval; SED, stent edge dissection.

## Discussion

### The main findings

The main findings in this study were as follows: 1) SED detected by OCT was observed in 18.3% of cases, 2) SED detected by OCT after current-generation DES implantation was significantly associated with MACE, and 3) Risk factors for SED detected by OCT were lumen eccentricity, stent-oversizing, and progressive atherosclerotic lesion (lipidic and fibrocalcific plaques).

### The incidence of SED detected by OCT in the current-generation DES era

Each imaging modality such as angiography, IVUS and OCT leads to different incidences of SED due to its resolution. Previous studies showed that SED was observed more often with IVUS (7.8–19.0%) than with angiography (0–4.8%) [1, 2, 24]. OCT can detect SED that IVUS may miss, since OCT has approximately 10 times better resolution than IVUS. Chamie et al. reported that only 16.0% of SEDs by OCT were detected by angiography alone [13]. Moreover, Bouma et al. showed that the prevalence of SED was higher in OCT (19.0%) versus IVUS (4.8%) in the same SEDs [25]. SED detected by OCT has been observed in 19.0% to 39.1% after stent implantation [9, 13, 25]. In the present study, 18.3% of patients underwent OCT-guide PCI had SED, which is concordant with previous OCT studies. Additionally, our study confirmed that SED more frequently occurred almost twice in the distal edges relative to the proximal edges (65.7% vs. 34.3%) [4, 13, 26].

### Relationship between SED and clinical outcomes after current-generation DES implantation

Previous studies using IVUS and angiography showed SED resulted in a higher rate of stent thrombosis and TLR, although SED had a high possibility of natural healing [2–5, 14, 27]. The OCT studies are more likely to include not only obvious but also small SED that IVUS or angiography cannot detect. There were several reports about whether SED detected by OCT including small dissection leads to worse clinical outcomes. Recently, in line with previous studies using IVUS and angiography, only a few studies reported that SED detected by OCT after stent implantation was associated with worse clinical outcomes, although earlier studies failed to show this significant association probably due to small number of patients [10, 11, 13, 14]. The previous OCT study reported by Prati et al. showed that distal dissection (>200μm) but not proximal dissection was one of predictors of MACE [10]. Moreover, only a few studies showed the predictors of MACE in patients with SED detected by OCT [9, 12]. The study reported by van Zandvoort et al. including 295 patients with SED showed that a predictor of MACE was only length of dissection [12]. All these studies included BMS and first-generation DES or did not show the prevalence of stents. Therefore, there was no study which focused on only current-generation DES.

Our question was whether these results can be simply applied to the current-generation DES. The current-generation DES equipped with biocompatibility improved safety and efficacy as compared to first-generation DES and BMS [15, 16]. A benefit of the current-generation DES might have generated an expectation for tolerable clinical outcomes of SED cases compared with non-SED cases, which was not observed in cases after first-generation DES or BMS implantation. However, in accordance with previous OCT studies, the present study showed significantly worse clinical outcomes in the SED group than in the non-SED group after current-generation DES implantation. Therefore, our results suggest that avoiding SED would be an important strategy even in the current-generation DES era.

### Risk factors for SED after current-generation DES implantation

In this study, progressive atherosclerotic plaques, i.e, lipidic and fibrocalcific plaques, and lumen eccentricity were the risk for SED after current-generation DES implantation, which was concordant with previous reports. Several previous studies showed the factors of SED after stent implantation such as excessive stent expansion, calcified or lipidic plaques, residual plaque eccentricity, stent length, and ST-elevation MI presentation [5, 13, 24]. Stenting on significant plaque such as fibrocalcific or lipidic plaque has been a well-known determinant of SED [5, 13, 24]. In these plaques, high tensile stress is generated at the junction between tissue types with differing elastic properties [28]. In the present study, lumen eccentricity was another predictor of SED. In a lesion with large lumen eccentricity, stenting causes unequal tensile stress in the same cross-section. Higher tensile stress occurs in the direction with a shorter lumen diameter than a longer one when the stent is expanded.

### Clinical implications

The previous studies have consistently demonstrated that small minimal stent area (MSA) significantly showed worse TLR and MACE [29]. Therefore, more aggressive post-dilatation especially in vessels with small MSA might be encouraged to achieve better stent expansion [8]. However, aggressive post-dilatation around stent edges to achieve optimal stent expansion might rather have the risk of SED resulting in worse clinical outcomes. The appropriate location of post-dilatation should be carefully chosen to avoid SED. Furthermore, even if the appropriate location was selected, there would be the possibility that post-dilatation affects the unplanned location due to insufficient visibility of stent or heart beat. Therefore, it might be acceptable to choose a downsized balloon when a balloon after stent implantation is in contact with a plaque which is not protected by a stent cage.

This study showed that progressive atherosclerotic plaques (lipidic and fibrocalcific plaques) by OCT and lumen eccentricity were associated with SED. Therefore, operators have to avoid locations with these features as stent landing zones. However, even though using OCT, SED related to stent-oversizing could not be completely avoided. One of the potential reasons for SED related to stent-oversizing is that the actual stent landing zones were different from those initially planned. Those unplanned stent landing zones can be led by heart-beat, longer or shorter length of the stents than planned, or any technical mistakes. Therefore, the possibility of stent landing at unplanned zones should be considered when stent length is selected. A choice of a downsized stent is also an option to avoid SED. When it is difficult to expect the landing zone, a downsized stent can be a choice for safety. The actual landing zone would be checked by an intra-coronary imaging device after an implantation of a downsized stent, and then, the appropriate size of post-balloon should be decided to correct stent malapposition if present.

### Study limitation

This study has the following limitations. First, this study was a retrospective and observational study at a single center. Second, the patient background might not be completely adjusted. The prevalence of ACS, which significantly differed between the SED and non-SED groups, was not included as the covariant set for IPTW, because the definition of AHA/ACC lesion classification includes the information of thrombus which is more frequently found in ACS than in stable angina. In the process of variables selection, if clinically similar variables remained, we selected the variable that we considered to be more clinically relevant in order to avoid multicollinearity. Therefore, AHA/ACC type classification was used as the covariant set rather than ACS or not. Third, the population of this study is small. Finally, this study covered the long study-period from 2010 to 2020, which was long enough to change the strategy of the procedure in a single center. In fact, changes in the PCI-strategy and -procedure might affect the occurrence of SED. However, those changes would not affect so much on the main conclusions of this study, since the aim of this study was to investigate the factors of SED and compare clinical outcomes between the SED and non-SED groups. However, in order to exclude possible confounding factors, further prospective and large-scale trials which include enough population during a short period are needed to assess the significance of SEDs.

## Conclusions

SED detected by OCT after current-generation DES implantation was associated with worse clinical outcomes. Atherosclerotic lesions, lumen eccentricity, and stent-oversizing were predictors of SED. The operators need to plan strategies to avoid SED, although it is important to achieve optimal stent expansion in order to improve clinical outcomes.

## Supporting information

S1 Dataset (XLSX)
